# Treatment outcomes with radium-223 in docetaxel-naïve versus docetaxel-treated metastatic castration-resistant prostate cancer patients: Real-world evidence from Taiwan

**DOI:** 10.1097/MD.0000000000032671

**Published:** 2023-02-03

**Authors:** Ping-Chia Chiang, Po-Hui Chiang, I-Hsuan Alan Chen, Yen-Ta Chen, Hung-Jen Wang, Yuan-Tso Cheng, Chih-Hsiung Kang, Chien-Hsu Chen, Yi-Yang Liu, Yu-Li Su, Yen-Hao Chen, Hao-Lun Luo

**Affiliations:** a Department of Urology, Kaohsiung Chang Gung Memorial Hospital and Chang Gung University College of Medicine, Kaohsiung, Taiwan; b Jhong Siao Urological Hospital, Kaohsiung, Taiwan; c Division of Urology, Department of Surgery, Kaohsiung Veterans General Hospital; d Department of Hematology and Oncology, Kaohsiung Chang Gung Memorial Hospital and Chang Gung University College of Medicine, Kaohsiung, Taiwan.

**Keywords:** adverse effects, alkaline phosphatase, castration-resistant prostatic neoplasm, docetaxel, radium-223

## Abstract

While radium (Ra)-223 is among the multiple, known life-prolonging treatments in bone-predominant metastatic castration-resistant prostate cancer (mCRPC), optimal treatment sequencing has not been determined, particularly in the Asia-Pacific context. Hence, we aimed to compare treatment outcomes of docetaxel-naïve and post-docetaxel mCRPC patients undergoing Ra-223 therapy in Taiwan. Using a single-center retrospective cohort design, we reviewed records of adult patients receiving Ra-223 for bone-metastatic mCRPC from 2018 to 2021. Patients were categorized into docetaxel-naïve or post-docetaxel groups based on history of docetaxel use preceding Ra-223. We compared the 2 groups in terms of all-cause death, 6-cycle treatment completion, and the following secondary outcomes: pain control, change in biochemical parameters (prostate-specific antigen, lactate dehydrogenase, alkaline phosphatase), biochemical response, and treatment-emergent adverse events. We performed total population sampling and a complete case analysis. We included 48 patients (25 docetaxel-naïve, 23 post-docetaxel) in the study. The mean follow-up duration was 12.4 months for the entire cohort. The docetaxel-naïve group exhibited a significantly lower all-cause mortality rate versus the post-docetaxel group (40.0% vs 78.3%, *P* = .02), as well as a significantly higher treatment completion rate (72.0% vs 26.1%, *P* < .01). We did not find significant differences in pain control, change in biochemical parameters, biochemical response, or hematologic treatment-emergent adverse events between the 2 groups. However, the docetaxel-naïve group had a numerically higher pain control rate, numerically greater improvements in alkaline phosphatase and prostate-specific antigen, and numerically lower rates of grade ≥ 3 neutropenia and grade ≥ 3 thrombocytopenia than the post-docetaxel group. Use of Ra-223 in docetaxel-naïve patients with mCRPC led to lower mortality and higher treatment completion than post-docetaxel use. Our study adds preliminary real-world evidence that Ra-223 may be used safely and effectively in earlier lines of treatment for bone-predominant mCRPC. Further large-scale, longer-term, and controlled studies are recommended.

## 1. Introduction

In 2020, prostate cancer (PC) was diagnosed in 7.3% of the global population, becoming the most common cancer in 112 countries and the second leading cancer in males worldwide.^[1]^ The mortality rate in patients with the localized disease has been characteristically low (i.e., < 10% in 30 years),^[2]^ yet in the presence of metastases, only ~30% have survived over 5 years, despite the emergence of new therapeutic options.^[3]^ Hence, PC has been the top cause of male cancer deaths in 48 countries across the globe.^[1]^

Bone metastases, developing in 80% to 100% of patients with advanced disease, has reduced 5-year survival rates in PC to < 20%.^[4]^ Alkaline phosphatase (ALP), a bone-related biochemical marker, is a prognostic factor in metastatic PC,^[5]^ along with the number and location of bone metastases.^[6]^ Meanwhile, complications of bone metastases, such as vertebral collapse, pathologic fracture, and spinal cord compression, have also contributed to significant morbidity in PC.^[6]^

As an *α*-emitting radionuclide with a short track length, radium-223 (Ra-223) induces double-strand breaks in metastatic bone lesions while causing minimal damage to surrounding normal tissue.^[7]^ Its clinical benefits in the treatment of metastatic castration-resistant prostate cancer (mCRPC) were established in ALSYMPCA, an international Phase III randomized controlled trial (RCT), wherein Ra-223 significantly improved overall survival (OS), symptomatic skeletal event-free survival, and quality of life versus placebo.^[8–10]^ In current guidelines, Ra-223 is a life-prolonging treatment indicated for adults with CRPC, symptomatic bone metastases and no known visceral metastases.^[6,11–14]^ However, its position in the optimal sequencing of therapies is still an ongoing debate.^[15]^

The Asia-Pacific region has exhibited a more advanced disease presentation of PC and a 3-fold higher mortality-to-incidence ratio compared with North America.^[16,17]^ Furthermore, treatment choices are influenced by distinct Asian treatment responses (e.g., increased toxicity to docetaxel, pharmacogenomic differences in testosterone metabolism), healthcare costs, and access issues.^[15,17]^ Thus, local research is warranted to formulate context-specific guidelines,^[17]^ especially with regards to treatment sequencing. Accordingly, we conducted this study to compare outcomes of docetaxel-naïve and post-docetaxel mCRPC patients treated with Ra-223 in Taiwan.

## 2. Materials and methods

### 2.1. Study design

We conducted a retrospective cohort study of mCRPC patients treated with Ra-223 in Kaohsiung Chang Gung Memorial Hospital, which caters to approximately 260 newly diagnosed PC patients in Southern Taiwan annually. In our institution, Ra-223 became available to patients beginning 2018. Accordingly, we reviewed electronic patient records from April 1, 2018 to December 31, 2021.

Ra-223 was prescribed in routine clinical practice at the discretion of the attending physician and administered intravenously at a standard dose of 55 kBq/kg every 4 weeks for up to 6 cycles. The concurrent use of androgen receptor-targeted agents (ARTA; abiraterone/enzalutamide) was allowed in this study. This study was approved by the Chang Gung Medical Foundation Institutional Review Board (IRB number: 202201111B0) and complied with the Declaration of Helsinki and the Medical Care Act of Taiwan. Although informed consent for study participation was waived because of the retrospective design, all data were de-identified to maintain confidentiality and data privacy.

### 2.2. Study population

Patients aged ≥ 18 years and diagnosed with bone-metastatic mCRPC were eligible for inclusion if they had received ≥1 cycle of Ra-223 during the study period. Presence of bone metastases was defined as ≥ 1 lesion on skeletal scintigraphy or magnetic resonance imaging while the definition of castration-resistant disease was based on contemporary European guidelines.^[6]^ We excluded patients who had visceral or maximal nodal metastases equal to or more than 3 cm in diameter, or underwent concurrent combination therapy with Ra-223 and docetaxel.

The first cycle of Ra-223 was set as the index period. Patients with a history of docetaxel use (i.e., any time before the index period) were categorized into the post-docetaxel group. Otherwise, they were classified under the docetaxel-naïve group. Patients who had been treated in the pre-index period with systemic chemotherapy other than docetaxel were excluded.

### 2.3. Variables and outcome measures

We collected the following baseline data from the pre-index period: age, Eastern Cooperative Oncology Group performance status (PS), Gleason score, bone metastases count, prior or concomitant use of ARTAs, hematologic parameters (hemoglobin, neutrophil count, platelet count), pain score, prior opioid use, and morphine equianalgesic dose. Data on biochemical parameters were gathered at baseline and every 4 weeks: ALP, prostate-specific antigen (PSA), and lactate dehydrogenase (LDH). We also noted the number of Ra-223 cycles received, any change in analgesic dose, any treatment-emergent adverse event (TEAE) based on the Common Terminology Criteria for Adverse Events version 3.0, and the occurrence of death from any cause.

#### 2.3.1. Primary outcomes of interest were.

1. All-cause death over the study period and;
2. Treatment completion, defined as completing 6 cycles of Ra-223. Secondary outcomes included pain control, change in biochemical parameters from baseline, biochemical response, and TEAEs. Change in PSA, LDH, or ALP referred to the percentage difference between baseline value and lowest value in the post-index period (i.e., nadir). A nadir lower than or equal to the baseline value constituted a biochemical response. We also explored a cutoff based on the ALSYMPCA trial: > 30% reduction from baseline to nadir.^[18]^ Meanwhile, pain control was defined as the absence of any increase in morphine equianalgesic dose from baseline to post-index period. In terms of TEAEs, we focused on grade ≥ 3 hematologic TEAEs.

All data were extracted from patient records by independent data collectors using standard data abstraction forms.

### 2.4. Statistical analysis

We used total population sampling in this study. Patients with missing data (e.g., patients lost to follow-up) were excluded in a complete case analysis. Missing data were assumed to be missing completely at random.

We summarized the categorical variables as proportions and the continuous variables as means with standard deviations. For categorical variables, the Chi-square test was used to compare the docetaxel-naïve and post-docetaxel groups while for continuous variables, we used the most appropriate among the *t*-test, ANOVA, Wilcoxon signed-rank test, or Kruskal-Wallis test. A *P*-value ≤ 05 was considered statistically significant. All statistical analyses were performed using R software version 4.1.2. (R Foundation for Statistical Computing, Vienna, Austria) We created waterfall charts with SAS^®^ OnDemand (version 9.4, SAS Institute Inc., Cary, NC) for Academics for visualization of changes in PSA, LDH, and ALP.

## 3. Results

**Figure 1.**
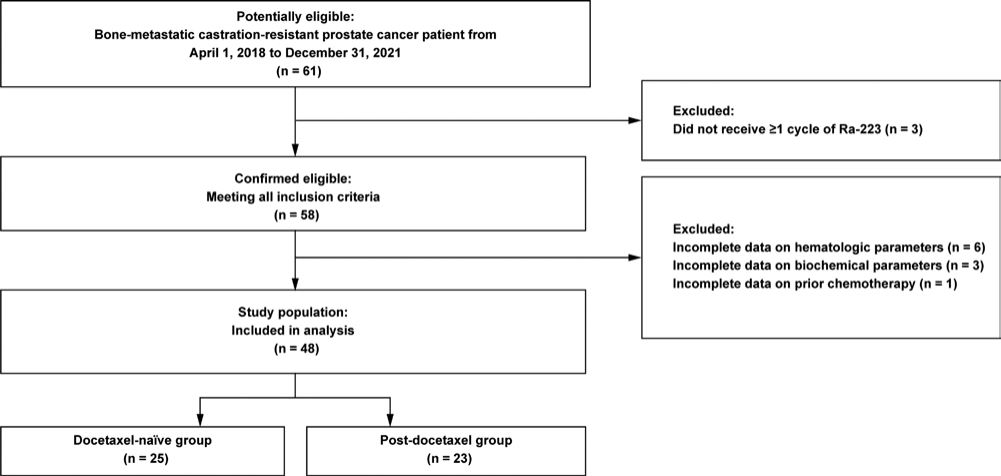
Patient flow diagram.

Out of 61 potentially eligible patients with bone-metastatic mCRPC, 3 patients did not receive ≥ 1 cycle of Ra-223 while another 10 patients had missing data. Hence, 48 patients were included in the analysis: 25 patients in the docetaxel-naïve group and 23 patients in the post-docetaxel group (Fig. 1).

At baseline, our total study population had a mean age of 73.8 ± 9.0 years and predominantly high-grade PC with a mean Gleason score of 8.6 ± 1.0 and mean PSA of 517.9 ± 1146.5 ng/mL. The docetaxel-naïve and post-docetaxel groups had no significant differences in baseline age, PS, tumor characteristics, ARTA use, pain and analgesia status, and hematologic parameters. LDH was significantly lower in the docetaxel-naïve group versus the post-docetaxel group (mean 231.8 U/L vs 313.2 U/L, *P* = .05), but the 2 groups were not significantly different in terms of other biochemical parameters at baseline (Table 1).

**Table 1:** Baseline characteristics of study subjects (N = 48).

Characteristic	Docetaxel-naive	Post-docetaxel	*P* value
	(n = 25)	(n = 23)	
Age (yr), median (IQR)	74 (13)	73 (12)	.34
Gleason score > 8, n (%)	21 (84.0)	19 (82.6)	1.00
Bone metastasis count < 6, n (%)	4 (16.0)	3 (13.0)	1.00
Prior or concomitant ARTA use, * n (%)	4 (16.0)	3 (13.0)	1.00
Prior opioid use, ** n (%)	15 (60.0)	12 (52.2)	.77
ECOG performance score, median (IQR)	1 (0)	1 (0)	.48
Pain score, † median (IQR)	3 (2)	4 (2)	.71
Morphine equianalgesic dose (mg), median (IQR)	5 (10)	5 (12)	.92
Hematologic parameters			
Hemoglobin (g/dL), median (IQR)	11.4 (1.4)	11.5 (1.3)	.89
Neutrophil count (per mm^3^), median (IQR)	3549.6 (2869.8)	4292 (3360.7)	.49
Platelet count (per mm^3^), median (IQR)	1,95,000.0 (83,000)	2,42,000 (1,18,500)	.03
Biochemical parameters			
PSA (ng/mL), median (IQR)	30.7 (293.2)	159.2 (331.3)	.25
ALP (U/L), median (IQR)	118 (212)	128 (330)	.70
LDH (U/L), median (IQR)	192 (113)	245 (199.5)	.07

* Abiraterone or enzalutamide.

** Opioids included: morphine, tramadol, fentanyl, oxycodone.

† Pain was measured using visual analog scale.

ALP = alkaline phosphatase, ARTA = androgen receptor targeted agent, ECOG = Eastern Cooperative Oncology Group, IQR = interquartile range, LDH = lactate dehydrogenase, PSA = prostate-specific antigen, SD = standard deviation.

The mean follow-up duration for the entire study cohort was 12.4 ± 9.1 months (docetaxel na*ï*ve: 12.6 ± 8.9 months, post-docetaxel: 12.2 ± 9.6 months). On average, our study patients received 4.7 ± 1.5 cycles of Ra-223 (docetaxel-naïve: 5.2 ± 1.3 cycles, post-docetaxel: 4.0 ± 1.5 cycles) during the study period.

### 3.1. Treatment outcomes

**Figure 2.**
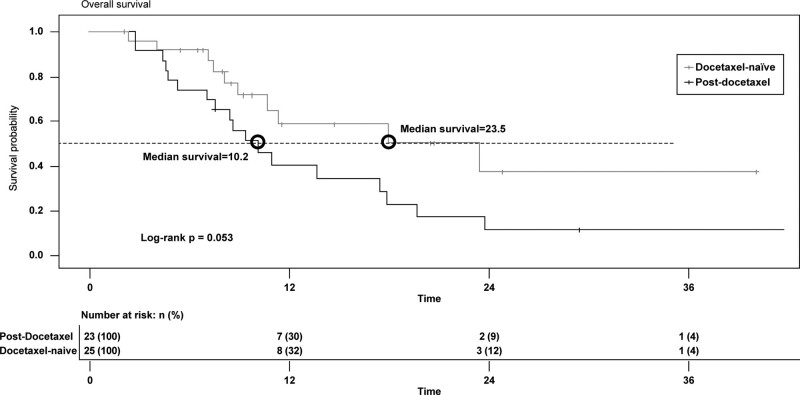
Kaplan–Meier analysis of overall survival among docetaxel-naïve and post-docetaxel patients receiving radium-223.

Table 2 shows the treatment outcomes in each study group. Over the study period, the docetaxel-naïve group exhibited a significantly lower all-cause mortality rate versus the post-docetaxel group (40.0% vs 78.3%, *P* = .02), as well as a significantly higher treatment completion rate (72.0% vs 26.1%, *P* < .01). Kaplan–Meier analysis shows a borderline difference of the overall survival (median 23.5 months vs 10.2 months, *P* = .053) between 2 groups (Fig. 2). Although pain control rate was numerically higher in the docetaxel-naïve group, the difference with the post-docetaxel group was not statistically significant (52.0% vs 30.4%, *P* = .27).

**Table 2:** Comparison of treatment outcomes between docetaxel-naïve and post-docetaxel patients receiving radium-223 (N = 48).

Outcome	Docetaxel-naïve	Post-docetaxel	*P* value
	(n = 25)	(n = 23)	
All-cause mortality, n (%)	10 (40.0)	18 (78.3)	.02
Treatment completion, * n (%)	18 (72.0)	6 (26.1)	< .01**
Pain control, § n (%)	13 (52.0)	7 (30.4)	.27
% ALP change, † median (IQR)	−44.7 (58.3)	−30.9 (20.9)	.23
ALP response, ‡ n (%)	21 (84.0)	22 (95.7)	.35
% PSA change, † median (IQR)	0 (118)	16.4 (71.4)	.35
PSA response, ‡ n (%)	13 (52.0)	9 (29.1)	.37
% LDH change, † median (IQR)	−2.4 ± 26.8	−10 ± 27.9	.09
LDH response, ‡ n(%)	15 (60.0)	17 (73.9)	.47

* Completed 6 cycles of Ra-223.

** *P* = .004.

† Percentage difference between baseline value and nadir in the post-index period.

‡ Nadir lower or < 5% higher than baseline value.

§ Absence of any increase in morphine equianalgesic dose from baseline to post-index period.

Definition of biochemical response: equal to or less than baseline.

ALP = alkaline phosphatase, LDH = lactate dehydrogenase, PSA = prostate-specific antigen, SD = standard deviation.

In terms of change in biochemical parameters, none of the differences between the 2 groups reached statistical significance (Table 2). However, compared with the post-docetaxel group, the docetaxel-naïve group had numerically greater improvements in ALP (mean change − 44.7% vs − 30.9%, *P* = .23) and PSA (mean change 0% vs 16.4%, *P* = .35) but lower improvements in LDH (mean change − 2.4% vs − 9.9%, *P* = .09). We found no significant differences in biochemical response rates between the 2 groups. In the exploratory analysis using > 30% reduction as cutoff (not shown in table), the docetaxel-naïve group had a numerically higher ALP response rate than the post-docetaxel group (64.0% vs 52.1%).

**Figure 3.**
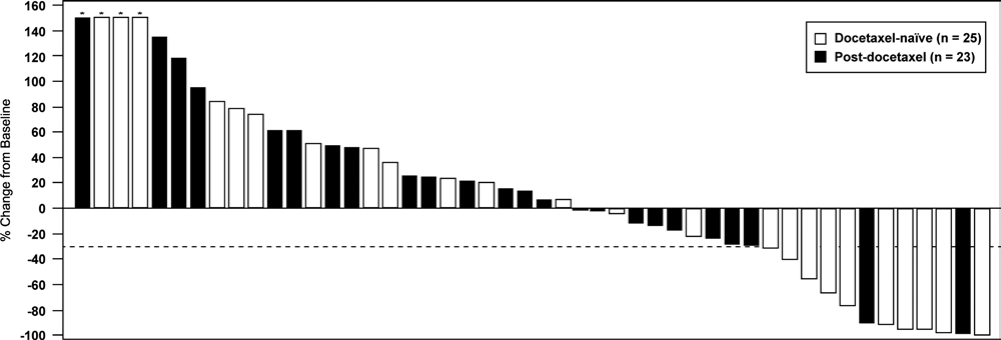
Waterfall plot showing percentage change in prostate-specific antigen from baseline to post-index nadir among docetaxel-naïve and post-docetaxel patients receiving radium-223 (N = 48). Dotted line marks ≥ 30% reduction. *Bars are higher than upper limit of graph.

**Figure 4.**
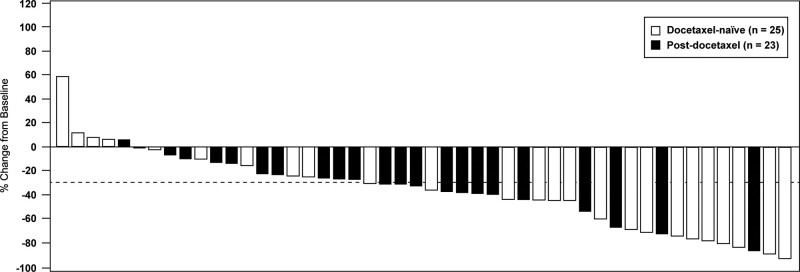
Waterfall plot showing percentage change in alkaline phosphatase from baseline to post-index nadir among docetaxel-naïve and post-docetaxel patients receiving radium-223 (N = 48). Dotted line marks ≥ 30% reduction.

**Figure 5.**
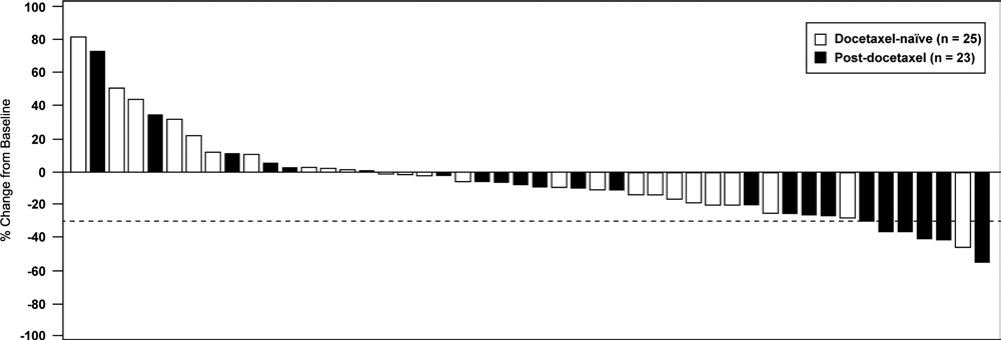
Waterfall plot showing percentage change in lactate dehydrogenase from baseline to post-index nadir among docetaxel-naïve and post-docetaxel patients receiving radium-223 (N = 48). Dotted line marks ≥ 30% reduction.

Figures 3–5 illustrate the changes in biochemical parameters from baseline among our study patients. Briefly, reductions in ALP were observed more frequently than reductions in PSA or LDH.

### 3.2. Adverse events

While rates of hematologic TEAEs were not significantly different between the 2 groups (Table 3), the docetaxel-naïve group displayed numerically lower rates of grade ≥ 3 neutropenia (0% vs 4.3%, *P* = .48) and grade ≥ 3 thrombocytopenia (4.0% vs 8.7%, *P* = .60) versus the post-docetaxel group.

**Table 3:** Comparison of rates of treatment-emergent adverse events between docetaxel-naïve and post-docetaxel patients receiving radium-223 (N = 48).

Treatment-emergent adverse event*	Docetaxel-naïve	Post-docetaxel	*P* value
	(n = 25)	(n = 23)	
Any grade ≥ 3 hematologic, n (%)	3 (12.0)	4 (17.4)	.70
Grade ≥ 3 anemia, n (%)	3 (12.0)	2 (8.7)	1.00
Grade ≥ 3 neutropenia, n (%)	0 (0)	1 (4.3)	.48
Grade ≥ 3 thrombocytopenia, n (%)	1 (4.0)	2 (8.7)	.60

* Using the common terminology criteria for adverse events version 3.0.

## 4. Discussion

In this real-world comparison of docetaxel-naïve and post-docetaxel mCRPC patients treated with Ra-223, we show that docetaxel-naïve patients had: (1) a significantly lower all-cause mortality rate and (2) a significantly higher treatment completion rate.

Our survival data mirror the findings from ALSYMPCA (N = 921), where median OS was numerically longer among docetaxel-naïve patients in the Ra-223 arm vs those previously treated with docetaxel (16.1 months vs 14.4 months). Additionally, docetaxel-naïve Ra-223 patients exhibited a numerically longer median time to a first symptomatic skeletal event than their post-docetaxel counterparts (17.0 months vs 13.5 months). However, in contrast to our study, ALP response rates (i.e., ≥ 30% reduction) were numerically comparable between docetaxel-naïve and post-docetaxel Ra-223 patients in the RCT (46% and 48%, respectively).^[18]^

Possible reasons for this disparity in findings include baseline differences between our cohort and the ALSYMPCA population. Compared with our study patients, the Ra-223 arm of the RCT had lower baseline PSA levels (median 146 mcg/L) and presumably less advanced disease. Asian race (3.6%) was also underrepresented in the RCT, possibly explaining the discrepancy with our study in terms of disease presentation. Moreover, the RCT had a higher treatment completion rate (65%) and less frequent deaths (54%) than our study, despite having a longer follow-up duration (3 years).^[8,19]^

In terms of adverse events, the occurrence of grade ≥ 3 myelosuppression was generally infrequent in the ALSYMPCA Ra-223 arm (21.5%),^[18]^ as it was in our study (18.8%). Post-docetaxel Ra-223 patients in the RCT had a significantly increased risk for grade ≥ 2 neutropenia or thrombocytopenia and numerically higher rates of grade ≥ 3 thrombocytopenia or pancytopenia than docetaxel-naïve Ra-223 patients.^[19]^ Our results illustrate an analogous numerical trend with regards to neutropenia or thrombocytopenia, but we would have possibly needed a larger sample size to reach the same statistical significance. Meanwhile, the RCT also demonstrated no significant association between previous docetaxel use and risk for grade ≥ 2 anemia.^[19]^ After high anemia rates were found in both treatment and placebo arms, the ALSYMPCA investigators surmised that the frequency and severity of anemia were influenced by disease burden rather than therapy.^[19]^

Apart from ALSYMPCA, previous Western real-world studies on Ra-223 use in mCRPC also support our findings. A prospective cohort study (N = 228) in the United Kingdom and a retrospective cohort study (N = 64) in the United States have both shown that prior chemotherapy (i.e., docetaxel and/or cabazitaxel) significantly predicted worse OS during Ra-223 therapy (*P* = .022 and *P* = .027, respectively).^[20,21]^ In the larger of these 2 studies, median OS was 12.3 months among chemotherapy-naïve patients vs 8.1 months among post-chemotherapy patients.^[20]^ Another retrospective cohort study (N = 220) in the United States found chemotherapy-naïve patients to have a significantly higher Ra-223 completion rate (76.6% vs 46.3%, *P* < .001) and lower discontinuation rates from disease progression (21.9% vs 37.8%, *P* = .039) or adverse events (3.1% vs 14.6%, *P* = .019) compared with post-chemotherapy patients.^[22]^

Multiple Asian real-world studies in this therapeutic area have been conducted in Japan.^[23–25]^ Corroborating our findings, a multicenter, prospective cohort study (N = 296) has shown numerically higher treatment completion rates and lower rates of grade ≥ 3 hematologic TEAEs among chemotherapy-naïve patients vs those with prior chemotherapy (i.e., docetaxel, cabazitaxel, or other).^[23]^ ALP and PSA response rates were also numerically higher in chemotherapy-naïve patients compared with post-chemotherapy patients, using either a cutoff of ≥ 30% reduction or any reduction.^[23]^ On the other hand, a smaller retrospective cohort study (N = 75) has reported that usage of Ra-223 at higher treatment lines was a significant predictor of treatment non-completion, which in turn was a significant predictor of poor OS.^[24]^ Similarly, another retrospective cohort study (N = 42) established a direct correlation between higher Ra-223 treatment lines and reduced OS.^[25]^

In our study, the higher treatment completion rate in the docetaxel-naïve group, which was almost thrice the value in the post-docetaxel group, possibly resulted in prevention or delay of disease progression, thus explaining the less frequent deaths among docetaxel-naïve patients. Collectively, the current real-world evidence, including our findings, suggests that earlier-line use of Ra-223 may benefit mCRPC patients by decreasing the risk of hematologic TEAEs and improving treatment completion, thereby reducing biochemical markers, and possibly prolonging overall survival.

Nevertheless, the optimal sequence of therapies in mCRPC has not been determined. In most guidelines, Ra-223 can be used as first-line therapy for mCRPC patients with symptomatic, bone-predominant metastases.^[11–14]^ Contrastingly, the European medicines agency (EMA) has limited Ra-223 use to post-ARTA therapy, after the ERA-223 trial had shown harm from the addition of Ra-223 to abiraterone.^[6]^ The EMA recommends that radium-223 be used for the treatment of adult patients with mCRPC, symptomatic bone metastases and no known visceral metastases, who are in progression after at least 2 prior lines of systemic therapy for mCRPC (other than luteinizing-releasing hormone analogues), or ineligible for any available systemic mCRPC treatment.^[6]^ Notably, in the 2019 Advanced Prostate Cancer Consensus Conference, 2-thirds of global experts did not agree with this EMA labeling.^[15]^ Likewise, our study reveals a low prevalence of prior or concomitant ARTA use (14.6%) among patients initiated on Ra-223 in routine practice. Despite this deviation from the EMA labeling, the mortality rate (40%) in our docetaxel-naïve Ra-223 group was comparable to the overall mortality rate (37%) in ERA-223.^[26]^ Hence, our results indirectly suggest that Ra-223 can be used safely and effectively before ARTA, contrary to EMA restrictions.

In Taiwan, Ra-223 was approved for use in 2015 and has been reimbursable under the National Health Insurance scheme since 2019.^[27,28]^ Nonetheless, local literature on the real-world experience with Ra-223 remains scant. A previous retrospective cohort study in Taiwan, albeit with a much smaller sample size (n = 18), found no significant difference in Ra-223 completion rates between chemotherapy-naïve and post-chemotherapy mCRPC patients.^[28]^ Reasons for non-completion in their study included poor PS, disease progression, and death while those in our study were mostly financial issues and loss to follow-up.^[28]^ On the other hand, as with our findings, pain control was not significantly different between the chemotherapy-naïve and post-chemotherapy groups.^[28]^ These conflicting results highlight the importance of conducting more local studies to expand available evidence, which could then inform future Taiwanese guidelines on optimal treatment sequencing in mCRPC.

Our study is limited by its retrospective, non-interventional, non-controlled design. While we could not completely remove confounders, the distribution of most baseline characteristics was not significantly different between the docetaxel-naïve and post-docetaxel groups. Our results are also unlikely to be affected by the baseline differences in LDH, which has not been predictive of outcomes in ALSYMPCA.^[9,19]^ However, our small single-center cohort, further constrained by missing data, may restrict the generalizability of our results. Lastly, we examined only intermediate outcomes with a short follow-up duration. Therefore, longer-term studies evaluating other clinically important endpoints (e.g., quality of life, skeletal events, OS) are warranted to confirm our results.

Notwithstanding these limitations, we show that the use of Ra-223 in docetaxel-naivety led to better treatment outcomes (i.e., lower mortality and higher treatment completion) compared with use after docetaxel. Accordingly, our study adds to the growing real-world evidence that Ra-223 may be used safely and effectively in earlier lines of treatment for symptomatic, bone-predominant mCRPC, particularly before chemotherapy with docetaxel. However, additional local studies are needed to clarify on optimal treatment sequencing in mCRPC in Taiwan.

## Acknowledgements

We thank the staff of the Kaohsiung Chang Gung Memorial Hospital, Kaohsiung Veterans General Hospital and Jhong Siao Urological Hospital for helping to care for the patients and to collect clinical information. Medical writing assistance was provided by Tristan Uy from and on behalf of MIMS, in compliance with Good Publication Practice 3 ethical guidelines (Battisti et al Ann Intern Med. 2015; 163: 461–4).

## Author contributions

**Conceptualization:** Ping-Chia Chiang, Po-Hui Chiang, Hao-Lun Luo.

**Data curation:** Ping-Chia Chiang, Po-Hui Chiang, I-Hsuan Alan Chen, Yen-Ta Chen, Hung-Jen Wang, Yuan-Tso Cheng, Chih-Hsiung Kang, Chien-Hsu Chen, Yi-Yang Liu, Yu-Li Su, Yen-Hao Chen, Hao-Lun Luo.

**Formal analysis:** Ping-Chia Chiang, Hao-Lun Luo.

**Funding acquisition:** Hao-Lun Luo.

**Investigation:** Hao-Lun Luo.

**Supervision:** Po-Hui Chiang.

**Writing-original draft:** Ping-Chia Chiang.

**Writing-review & editing:** Hao-Lun Luo.
